# Analysing the Role of UVB-Induced Translational Inhibition and PP2Ac Deactivation in NF-κB Signalling Using a Minimal Mathematical Model

**DOI:** 10.1371/journal.pone.0040274

**Published:** 2012-07-18

**Authors:** Johannes Witt, Fabian Konrath, Oliver Sawodny, Michael Ederer, Dagmar Kulms, Thomas Sauter

**Affiliations:** 1 Institute for System Dynamics, University of Stuttgart, Stuttgart, Germany; 2 Institute of Cell Biology and Immunology, University of Stuttgart, Stuttgart, Germany; 3 Life Sciences Research Unit, University of Luxembourg, Luxembourg, Luxembourg; Christian-Albrechts-University Kiel, Germany

## Abstract

Activation of nuclear factor κB (NF-κB) by interleukin-1β (IL-1) usually results in an anti-apoptotic activity that is rapidly terminated by a negative feedback loop involving NF-κB dependent resynthesis of its own inhibitor IκBα. However, apoptosis induced by ultraviolet B radiation (UVB) is not attenuated, but significantly enhanced by co-stimulation with IL-1 in human epithelial cells. Under these conditions NF-κB remains constitutively active and turns into a pro-apoptotic factor by selectively repressing anti-apoptotic genes. Two different mechanisms have been separately proposed to explain UV-induced lack of IκBα recurrence: global translational inhibition as well as deactivation of the Ser/Thr phosphatase PP2Ac. Using mathematical modelling, we show that the systems behaviour requires a combination of both mechanisms, and we quantify their contribution in different settings. A mathematical model including both mechanisms is developed and fitted to various experimental data sets. A comparison of the model results and predictions with model variants lacking one of the mechanisms shows that both mechanisms are present in our experimental setting. The model is successfully validated by the prediction of independent data. Weak constitutive IKKβ phosphorylation is shown to be a decisive process in IκBα degradation induced by UVB stimulation alone, but irrelevant for (co-)stimulations with IL-1. *In silico* knockout experiments show that translational inhibition is predominantly responsible for lack of IκBα recurrence following IL-1+UVB stimulation. In case of UVB stimulation alone, cooperation of both processes causes the observed decrease of IκBα. This shows that the processes leading to activation of transcription factor NF-κB upon stimulation with ultraviolet B radiation with and without interleukin-1 costimulation are more complex than previously thought, involving both a cross talk of UVB induced translational inhibition and PP2Ac deactivation. The importance of each of the mechanisms depends on the specific cellular setting.

## Introduction

The transcription factor nuclear factor κB (NF-κB) is of fundamental importance in anti-apoptotic signalling and inflammation, since it is activated by a multitude of stimuli, and causes a wide range of cellular responses. Constitutive NF-κB activation contributes to the maintenance of a variety of cancers by inducing expression of anti-apoptotic genes [1], [2]. Manifold strategies to fight cancer are therefore based on NF-κB inhibition [1], . In resting cells, almost all NF-κB resides in the cytosol and is kept inactive by its binding to the inhibitor of κBα (IκBα).

Canonical activation of NF-κB by pro-inflammatory cytokines, like interleukin-1β (IL-1), is mediated by a signalling cascade resulting in activation of the IκB kinase complex (IKK). In particular, its catalytic subunit IKKβ is phosphorylated at Ser177/181 [4] and subsequently phosphorylates the NF-κB inhibitor IκBα at Ser32/36. Accordingly, IκBα becomes polyubiquitinated and is proteasomally degraded, consequently liberating NF-κB. Free NF-κB translocates to the nucleus, where it activates the transcription of responsive genes, including the one encoding its inhibitor IκBα [4], [5]. Newly synthesised free IκBα translocates to the nucleus where it binds to NF-κB, thus terminating NF-κB activity and initiating nuclear export of the complex. Since NF-κB dependent genes account for proliferation, inflammation, and anti-apoptosis, this autoregulatory negative feedback loop ensures proper cellular function. Dysregulation causes constitutive NF-κB activation, which is linked to maintenance of a variety of cancers as well as chemo resistance [1], [2].

The complex feedback behaviour of the NF-κB signalling as well as the importance of this behaviour in cellular systems have given rise to a variety of deterministic and stochastic mathematical models [6]. These models crucially aided in the understanding of many of the fascinating details of this signalling system, e.g. the role of the different IκB isoforms [7],[8], the influence of IKK on NF-κB responses [9], [10], the regulation of IKK phosphorylation [11], [12], [13], [14], single cell dynamics [15], [12], cross-talk mechanisms [16], the balance of life/death decisions [17] or the emergence and potential role of oscillations [12], [18], [19].

In contrast to the established role of NF-κB as an anti-apoptotic factor, we and others revealed that NF-κB becomes a mediator of pro-apoptotic responses when combined with certain DNA damaging agents. Co-stimulation of human epithelial cells, or keratinocytes, with ultraviolet B (UVB) radiation and IL-1 resulted in enhancement of the UVB-driven apoptotic response via NF-κB-dependent repression of anti-apoptotic genes [20], [21], [22], [23]. The final outcome of NF-κB behaviour thereby appeared to depend on the nature of the DNA damage induced [24]. These observations demand for a detailed investigation of the underlying mechanisms, because they may alter the individual strategy for combination therapies.

Strikingly, enhancement of apoptosis in UVB+IL-1 treated cells was associated with complete disappearance of the negative feedback loop of NF-κB through inhibition of IκBα recurrence, consequently causing sustained NF-κB activation [25]. While others reported inhibition of IκBα resynthesis in UVC treated mouse embryonic fibroblasts (MEF) exclusively resulting from translational inhibition [16], we revealed a more complex mechanism following UVB+IL-1 stimulation. Using experimental approaches as well as a strictly reduced mathematical model, we showed that the catalytic subunit of the Ser/Thr phosphatase PP2A (PP2Ac) becomes deactivated upon UVB radiation, causing incomplete IKKβ dephosphorylation. Chronically activated IKKβ consequently phosphorylates newly synthesised IκBα, marking it for proteasomal degradation, thus enabling prolonged NF-κB activation [25],[13].

Envisaging these two different mechanisms, the aim of the present study was to quantify the contribution and importance of UVB-induced translational inhibition compared to PP2Ac-initiated posttranslational IκBα degradation with regard to the elimination of the negative feedback loop of NF-κB. Using a systems biological approach, we coupled our previously developed IKKβ phosphorylation model [13] to a minimal NF-κB model. The model not only reproduces several experimental data sets, but in turn also produces reliable predictions. We show that unlike translational inhibition, UVB-induced PP2Ac deactivation is crucial for sustained NF-κB activity in human epithelial cells following IL-1+ UVB stimulation, while both processes are required to induce IκBα degradation following UVB irradiation alone.

## Results and Discussion

### Development of a Reduced Order Mathematical Model of NF-κB Signalling Including UVB-induced Effects

**Figure 1:**
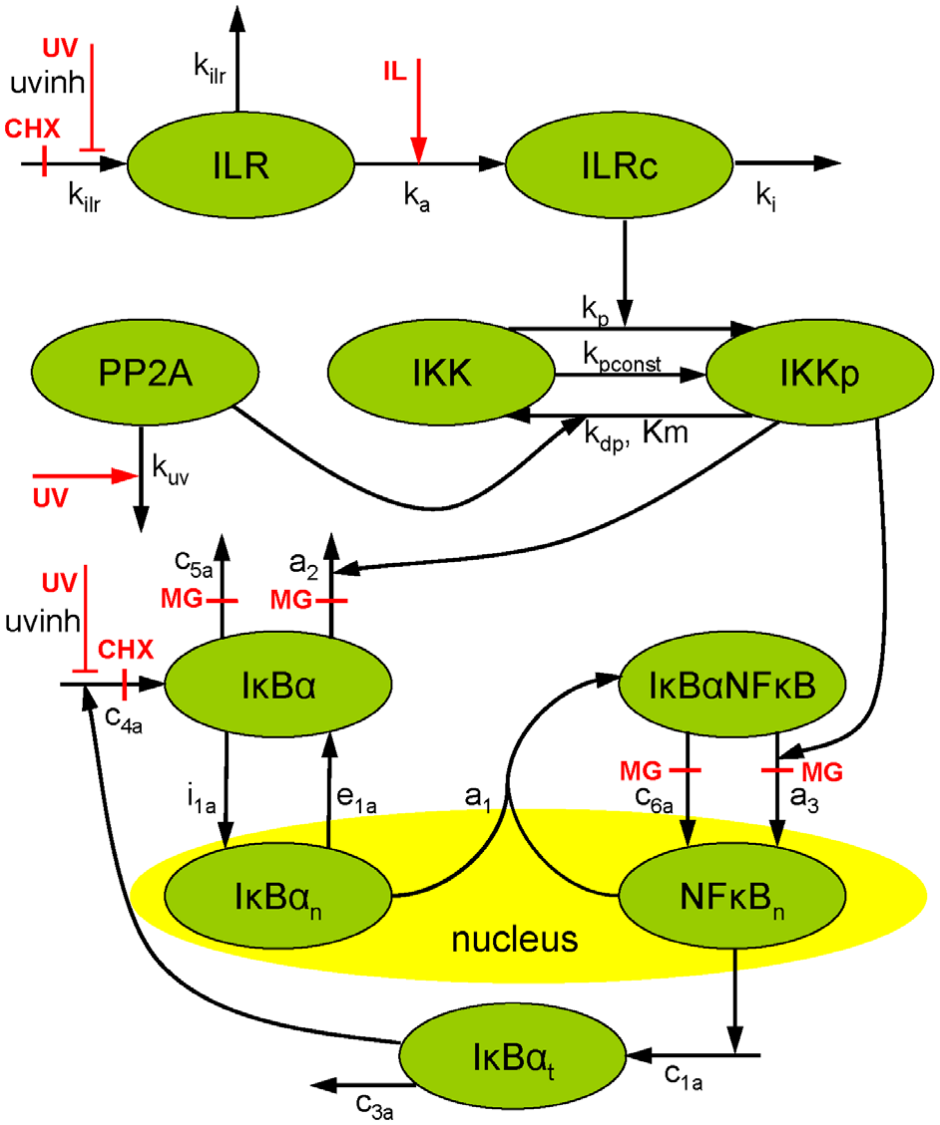
Schematic representation of the reference model. System variables are depicted in green, inputs are depicted in red. Abbreviations and parameter names are explained in Tables S1 and S2. The model variants lacking translational inhibition or PP2Ac deactivation are implemented by setting *uvinh* = 0 or *k_uv_* = 0, respectively.

In order to address the question of which UVB-induced mechanism is decisive in inhibiting IκBα recurrence, we designed a mathematical model of the relevant processes (model M_Ref_, Figure 1 and File S1). As described in detail in the *Material and Methods* section, our model comprises two parts, an adapted version of our recently published model of IKKβ phosphorylation [13] and a module describing the IκBα-NF-κB interactions, inspired by the model of Lipniacki et al. [26] and modified due to more recent findings (e.g. [16],[12]). Additionally, we applied model reduction strategies to further decrease the model size (see *Material and Methods*), as a smaller model tends to be more discriminative and less prone to overfitting, due to a lower number of degrees of freedom.

While M_Ref_ (Figure 1) includes both UVB-induced PP2Ac deactivation and translational inhibition, we also developed two model variants, one lacking UVB-induced PP2Ac deactivation (model M_P_) and the other lacking translational inhibition (model M_T_). We now strived to identify which of the models adequately describes the experimental data and to validate the model by independent experiments in an iterative process of modelling and experimental work.

### Lack of IκBα Recurrence is not Exclusively Due to UVB-induced Translational Inhibition

**Figure 2:**
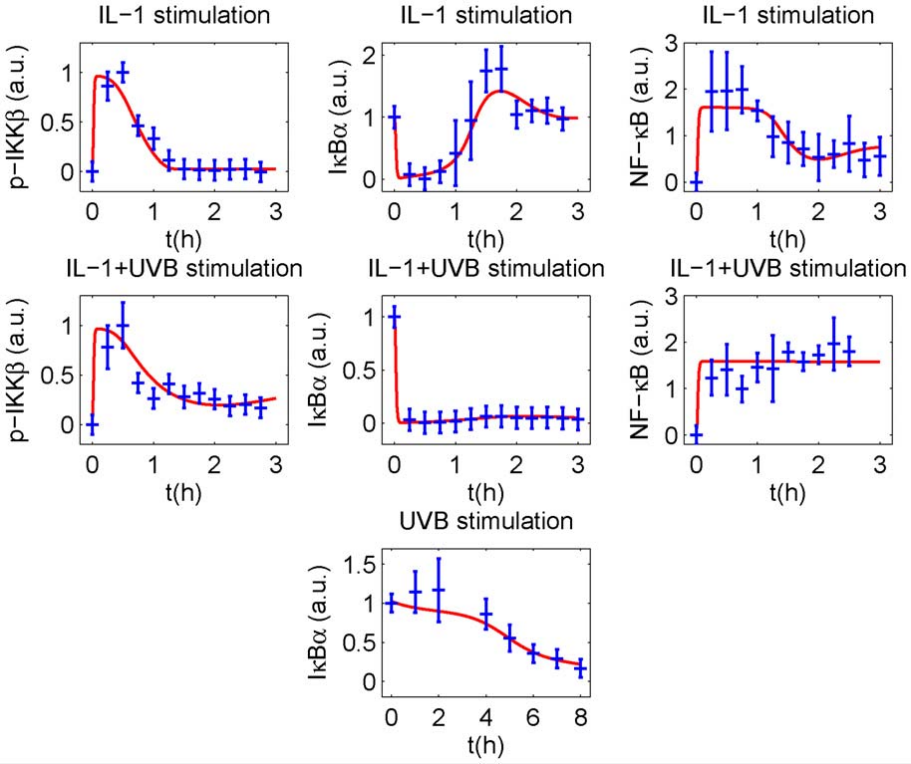
Model results in the reference model. Assuming both UVB induced PP2Ac deactivation and translational inhibition, the model convincingly reproduces the experimental data for IL-1 (10 ng/ml), IL-1 (10 ng/ml) + UVB (300 J/m^2^) and UVB (300 J/m^2^) stimulation. Experimental data and standard deviations (blue) of phosphorylated IKKβ, total cellular IκBα and nuclear NF-κB are compared to the results of the model (red) fitted to these data. The NF-κB time courses were scaled to a mean of 1 and subsequently averaged. IκBα Western blots were standardised to an initial value of 1.

Data from quantified Western blots and electrophoretic mobility shift analysis (Figure 2) show that following IL-1 stimulation, IKKβ is rapidly phosphorylated and subsequently dephosphorylated. Almost complete dephosphorylation is reached after about 75 min. IKKβ phosphorylation induces complete IκBα degradation, followed by its resynthesis yielding an overshoot after about 90 min. Accordingly, NF-κB activity increases rapidly and decreases to a low level after about 2 h. In contrast, combined IL-1 and UVB stimulation causes only incomplete dephosphorylation of IKKβ, and NF-κB remains active because IκBα does not recur. Upon UVB stimulation alone, IκBα degradation follows much slower kinetics.

All models were fitted to the measured time courses, minimising the χ^2^ value.

The models M_Ref_ and M_T_ convincingly reproduced the experimental data, reflected by χ^2^ values of 38.1 and 39.3, respectively (Figures 2 and S1). In contrast, model M_P_ failed to reproduce the IκBα overshoot upon IL-1 stimulation and the sustained IKKβ phosphorylation upon IL-1+UVB stimulation (χ^2^ = 58.2, Figure S2). Therefore, we rejected the hypothesis that translational inhibition alone was responsible for the observed behaviours following UVB irradiation with and without IL-1 costimulation.

### PP2Ac Deactivation and Translational Inhibition Both Contribute to the Observed System Behaviour

**Figure 3:**
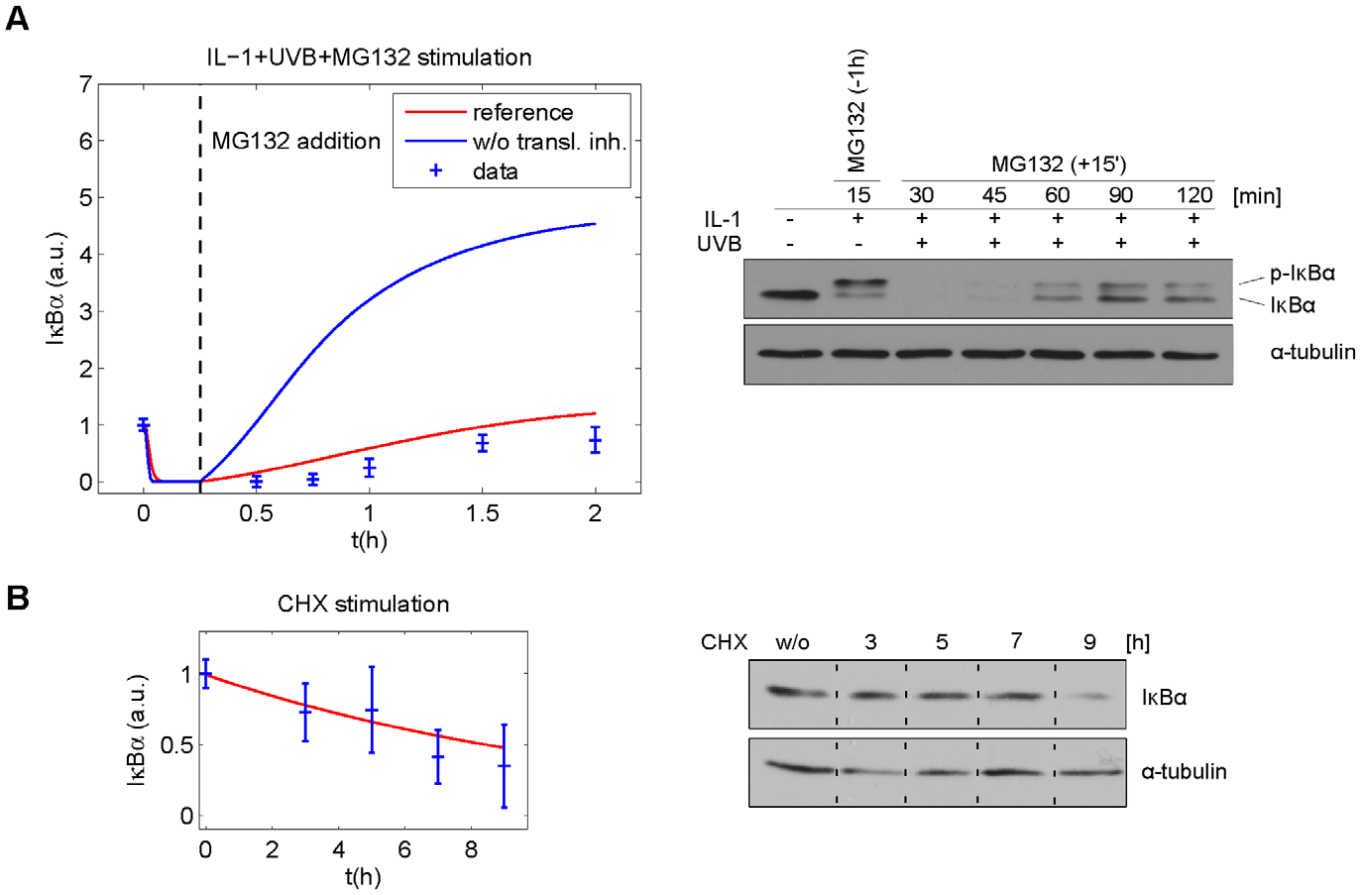
Model prediction and validation. (**A**) The models M_Ref_ and M_T_ predict markedly different IκBα concentrations following IL-1 and UVB stimulation and addition of proteasome inhibitor MG132 15 min post stimulation. The proposed experiment follows the concentration course predicted by the reference model. This confirms the hypothesis that both PP2Ac deactivation and translational inhibition are relevant processes for the lack of IκBα recurrence. KB cells were left untreated or stimulated with IL-1+UVB for the indicated time points. The proteasome inhibitor MG132 (MG) was applied 1 h in advance (−1 h) or 15 min after initial IL-1 stimulation (+15′). The status of IκBα protein was determined by Western blot analysis. Equal loading was monitored by reprobing the membrane with an anti α-tubulin antibody. The quantified blots were standardised to an initial value of 1. Parameter values were optimised using the data for IL-1, IL-1+UVB and UVB, but not for IL-1+UVB+MG132 stimulation. (**B**) Experimental predictions are perfectly in line with IκBα concentrations following CHX stimulation. KB cells were left untreated or stimulated with the translation inhibitor cycloheximide (CHX) for the indicated time points. The status of IκBα protein was determined by Western blot analysis. Equal loading was monitored by reprobing the membrane with an anti α-tubulin antibody. Protein bands shown are extracted from the same membrane under identical conditions. Parameter values were optimised using the data for IL-1, IL-1+UVB, UVB and IL-1+UVB+MG132 stimulation, but not for CHX stimulation.

Since the fit quality did not allow for discrimination between models M_Ref_ and M_T_, we used simulation studies to select an additional experiment for model discrimination: addition of the proteasome inhibitor MG132 15 min after IL-1+UVB stimulation, when IκBα degradation is completed, allows the investigation of IκBα synthesis without IκBα degradation. Accordingly, model M_Ref_ predicted a moderate IκBα increase to about the initial level, while model M_T_ predicted a 4 fold increase compared to the initial IκBα level (Figure 3A). The experimental data confirmed the prediction of model M_Ref_ but not the prediction of model M_T_ (Figure 3A).

In order to exclude that model M_T_ might be able to reproduce the experimental data if they were known *a priori*, we performed a new fit including the newly found experimental data. Still, model M_T_ could not convincingly reproduce the experimental data, while model M_Ref_ could, as reflected by χ^2^ values of 98.9 vs. 42.7 (Figures S3 and S4). Conclusively, both UVB-induced translational inhibition and PP2Ac deactivation contribute to the system behaviour.

### The Model Correctly Predicts Additional Experimental Data

Before investigating the contribution of each of the two mechanisms in M_Ref_, this model was further validated and analysed: in order to determine constitutive IκBα degradation, cells were stimulated with translation inhibitor cycloheximide (CHX). The observed slow decrease of IκBα with a half-life of about 6 h was well predicted by our model without any additional fit (Figure 3B). Subsequent fitting to all experimental data leaves the model almost unchanged (Figure S5, Table S1) and provides the basis for the further investigations. The similar values of the rate constants of free and NF-κB-bound IκBα, *a_2_* and *a_3_* (Table S1), represent a noteworthy detail additionally validating the model: they are in line with findings of Mathes et al. [27] who showed that free and NF-κB-bound IκBα have similar phosphorylation kinetics. Altogether, the various model validations show that the model can reliably be used as a starting point for further analysis and *in silico* experiments.

### Differences of the IκBα Degradation Rate Following UV Irradiation Can be Explained by Small Absolute Variations in Constitutive IKKβ Phosphorylation

**Figure 4:**
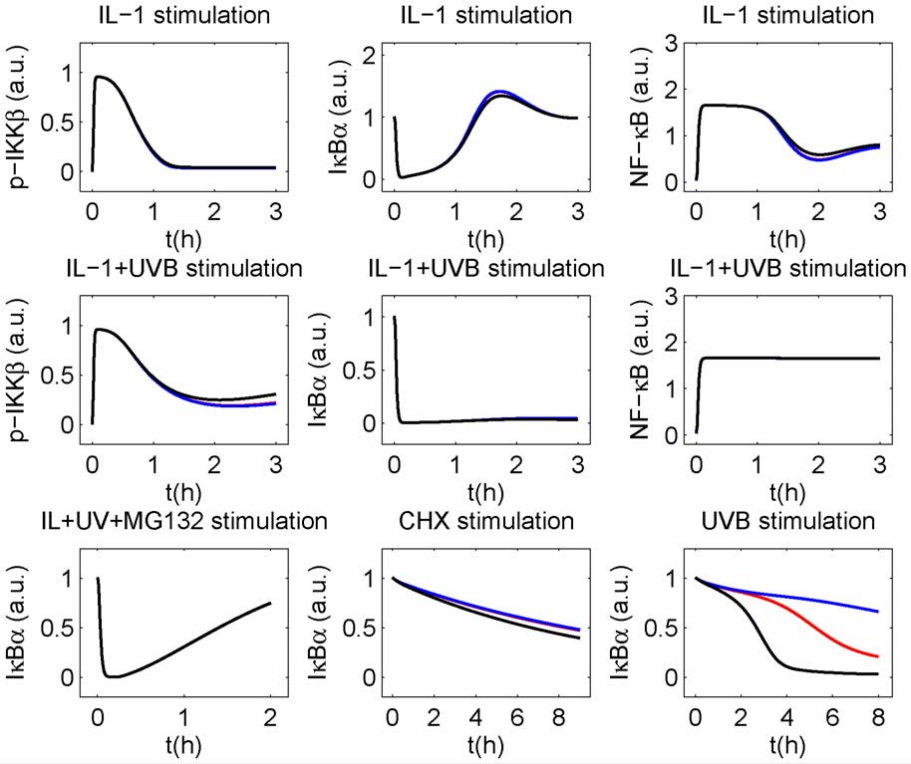
IκBα degradation upon UVB stimulation critically depends on constitutive IKKβ phosphorylation. Model results of model M_Ref_ for protein levels with *k_pconst_* as determined by fitting (red), ten fold larger *k_pconst_* (black) and ten fold lower *k_pconst_* (blue). For any stimulation except UVB stimulation alone, the differences are marginal.

While IκBα degradation following UVC irradiation seems to be complete after 2 h in mouse embryonic fibroblasts (MEFs) [16], IκBα degradation following UVB stimulation in our human epithelial KB cells started after 4 h and decreased to lower levels after 8 h (see Figure 2). Furthermore, even slower IκBα degradation following UVB stimulation was reported in earlier experiments with KB cells, with IκBα being still incompletely degraded 16 h after UVB stimulation [25]. Since a notable constitutive basal IKKβ activation level existed in the MEFs [16] but not in human KB cells [13], we investigated the impact of varying the parameter for constitutive IKKβ phosphorylation, *k_pconst_*, on IκBα degradation. While IκBα was completely degraded after 4 h for ten fold larger *k_pconst_*, only 60% of IκBα is degraded after 16 h for ten fold lower *k_pconst_*, indicating that variations of *k_pconst_* can easily explain the observed differences in IκBα degradation (Figure 4). Note that the value of *k_pconst_* is very low, so that the large relative changes only correspond to low absolute changes. It is well conceivable that small differences in the experimental conditions may be responsible for the very low absolute changes of basal IKKβ activation. This shows why even cell populations from the same cell line may react differently to UVB stimulation. Importantly, these changes remain almost unnoticed for the other investigated stimulations (Figure 4) because the effect of *k_pconst_* is easily counteracted by PP2Ac unless PP2Ac is deactivated by UVB radiation.

### PP2Ac Deactivation is the Predominant Mechanism in Inhibiting IκBα Recurrence Following IL-1+UVB

In order to investigate in more detail the lack of IκBα recurrence following IL-1+ UVB stimulation, we now compared the contributions of the individual processes, UVB induced translational inhibition and PP2Ac deactivation, to this behaviour. Up to this point, the obtained results seemed ambiguous: on the one hand, model M_T_ - but not M_P_ - could reproduce the experimental data for single and combined IL-1 and UVB stimulation (Figures S1 and S2). This suggests that PP2Ac deactivation has a large effect on IκBα inhibition. On the other hand, model M_Ref_ predicts an UVB-induced translational inhibition of about 90% (Table S1), indicating that translational inhibition may be predominant.

**Figure 5:**
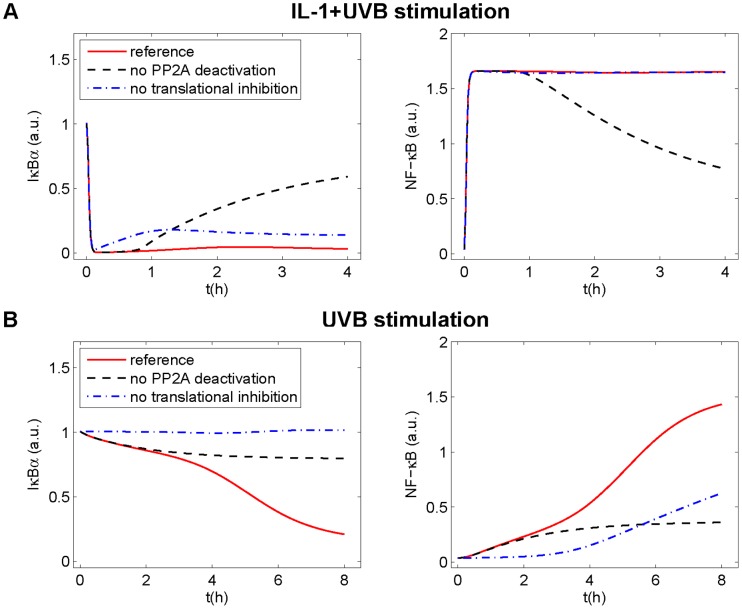
Relevance of UVB-induced translational inhibition and PP2Ac deactivation for IκBα and NF-κB upon UVB+/−IL-1 stimulation. Knocking out the processes of UVB-induced translational inhibition or PP2Ac deactivation *in silico*, their importance for IκBα and NF-κB in model M_Ref_ is determined. (A) Upon IL-1+UVB stimulation, lack of PP2Ac deactivation leads to recurrence of IκBα and decrease of NF-κB activity, while lack of translational inhibition only leads to a minor increase of the IκBα concentration. (B) Upon UVB stimulation, lack of PP2Ac deactivation leads to a modest decrease of IκBα concentration and to a modest increase of NF-κB activity, while lack of translational inhibition leads to a slow increase of NF-κB activity.

Since it is almost impossible to distinguish between both UVB effects in a cellular setting, we addressed the question by shutting off both effects separately *in silico*. Deletion of UVB-induced PP2Ac deactivation (*k_uv_ = *0) leads to IκBα recurrence and decrease of NF-κB activity. Strikingly, deletion of UVB-mediated translational inhibition (*uvinh = *0) only has a minor effect on IκBα recurrence and no effect on NF-κB activity (Figure 5A). In this context, it must be taken into account that translational inhibition was reported to occur through phosphorylation of the global translation factor eukaryotic initiation factor-2α (eIF2α) [28], [29]. Thus, UVB affects both IκBα and IL-1 receptor (ILR) synthesis in our model. As a consequence, translational inhibition not only reduces IκBα resynthesis, but - via reduction of ILR resynthesis - also signal transduction and downstream IKKβ phosphorylation. Accordingly, UVB-induced translational inhibition triggers two roughly balanced effects, one increasing and one decreasing the intracellular IκBα level. Without PP2Ac deactivation, however, almost total dephosphorylation of IKKβ would occur, preventing relevant IκBα degradation. In this scenario, the main effect of translational inhibition is an increase of the time constants, i.e. it prolongs the time until a steady state concentration is reached. In conclusion, both UVB-induced translational inhibition and PP2Ac deactivation are present following IL-1+UVB stimulation, but PP2Ac deactivation is clearly the most important mechanism for inhibition of IκBα recurrence.

### Cooperation of PP2Ac Deactivation and Translational Inhibition is Required to Cause Decrease of IκBα Following UVB Stimulation Alone

Interestingly, a different result is obtained for the contribution of UVB-induced translational inhibition and PP2Ac deactivation to IκBα decrease following stimulation with UVB alone.

Deleting both effects separately *in silico*, we found that neither PP2Ac deactivation nor translational inhibition alone is sufficient to induce a significant IκBα degradation (Figure 5B): without PP2A deactivation, only small amounts of IκBα are degraded so that efficient translation is not required. Without translational inhibition, phosphorylated IKKβ slowly accumulates, but in low concentrations so that an intact translational machinery can counteract this effect. Consequently, the NF-κB activity is much lower than when both processes are functional (Figure 5B). Others have reported translational inhibition to be uniquely responsible for UV-induced IκBα degradation in cells with a much higher level of constitutively phosphorylated IKKβ [16]. Our model also successfully reproduces this result with accordingly modified parameter values (Figure S6).

Summarising, in our setting the observed IκBα degradation following UVB stimulation requires a combination of both, translational inhibition and PP2Ac deactivation.

### Conclusions

We showed that our minimal model of the NF-κB signalling pathway is completely sufficient to describe NF-κB signalling following various stimulations including different combinations of IL-1, UVB, MG132, and CHX. The model quantitatively reproduces and predicts detailed kinetic data of three proteins in several experimental settings. It can be used as a reliable building block in future models of inflammation and apoptosis and is easy to handle due to its small size. We used the model to unravel the contributions of two UVB-induced mechanisms, namely global translational inhibition and PP2Ac deactivation, to the phenomenon of sustained NF-κB activity following IL-1+UVB stimulation and to IκBα degradation following UVB stimulation alone.

Our model finds that translational inhibition cooperates with PP2Ac deactivation in decreasing the cellular IkBa level upon UVB irradiation, while PP2Ac deactivation plays the predominant role in IL-1+UVB treated cells.

## Materials and Methods

### I) Mathematical Modelling

Our model consists of an adapted version of our IKKβ phosphorylation model [13] and an IκBα-NF-κB module based on the model of Lipniacki et al. [26] and recent findings (e.g. [16], [12]). As we showed recently, the decoupling of the IκBα-NF-κB module from the upstream part involving IKKβ is possible in the case of IL-1 stimulation [13].

Our previous model of IKKβ phosphorylation [13] was adopted with some slight modifications:

The parameter values were not fixed to the fitted values from the previous model [13]. However, for those parameters comparable to the previous model the fitted parameter values were very similar to those of the previous model (Table S1).
Since even small amounts of phosphorylated IKK can have significant effects on the downstream signalling, we incorporated IL receptor turnover. We can normalise the system such that the degradation rate constant can be used for both synthesis and degradation of the receptor without loss of generality ([13], supplemental material).
Furthermore, to account for the possibility that the amount of PP2Ac available for binding to the IKK complex limits IKKβ dephosphorylation we introduced a Michaelis-Menten term for the IKKβ dephosphorylation process.
Since constitutive IKKβ phosphorylation has been shown to be relevant for UVB-induced IκBα degradation [16], this process was also considered.

Note that in contrast to TNFα stimulation, receptor internalisation following IL-1 stimulation is fast [13].

The downstream part of the model is based on the model of Lipniacki et al. [26]. However, we simplified this model quite substantially:

As we previously showed [13], the state variables *IKKa|IκBα* and *IKKa|IκBα|NFκB* can be eliminated using the quasi-steady-state approximation, which only marginally affects the model output.
Furthermore, the A20 feedback loop does not play a decisive role for IL-1 induced NF-κB signalling [30], [13]. It was therefore removed from the model.
Nuclear export of IκBα-NF-κB complex is a very fast process [26], [12]. Therefore we applied the quasi-steady-state assumption to eliminate the state variable *IκBα_n_|NFκB_n_*. Nuclear import of NF-κB is slower, but still considerably fast (0.0026 s^−1^ [12]). Tentative model reduction using the quasi-steady-state assumption yielded an only marginally worse fit. We therefore also eliminated the state variable *NFκB*.
Like most NF-κB models (e.g. [7], [26], [12]), we assume mass conservation of NF-κB, i.e. NF-κB is neither synthesised nor degraded in our model. This allows the elimination of another differential equation. We choose the differential equation describing the temporal change of *IκBα|NFκB*.

The effects of the different stimulations are the following: CHX inhibits ILR and IκBα synthesis. Note that CHX has no effect on IKK, PP2A and NF-κB in our model, because we assume that synthesis of these proteins does not occur in relevant amounts at the considered time scales. IL-1 induces ILR complex formation, MG132 terminates proteasomal degradation of IκBα, i.e. both IKKβ dependent and independent degradation are inhibited [27]. Potential effects of MG132 on protein synthesis [31] were not considered. UVB radiation attenuates IκBα synthesis [16] by a (fitted) fraction *uvinh*. Since translational inhibition was reported to occur through phosphorylation of eukaryotic initiation factor-2α (eIF2α) [28], [29], we assumed that the same fraction *uvinh* of ILR synthesis is affected. eIF2α phosphorylation occurs almost immediately following UVB stimulation [32] so that a constant UVB-induced translational inhibition is assumed.

Taken together, the model equations of the reference model (Figure 1) read
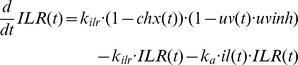


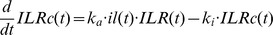


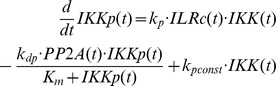


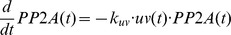


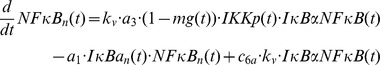


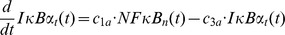


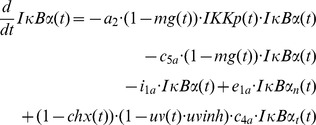


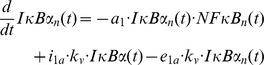
where *IKK*(*t*) = 1– *IKKp*(*t*) and *IκBαNFκB*(*t*) = *nfkb_tot_* – *NFκB_n_*(*t*)/*k_v_*, as obtained from the mass conservation of IKK and NF-κB, respectively. *nfkb_tot_* is the concentration of NF-κB if it is completely in the cytoplasm. Division by the cytoplasm to nucleus volume ratio *k_v_* is required to account for the different compartments of *IκBαNFκB*(*t*) and *NFκB_n_*(*t*). Tables S1 and S2 describe the meaning of the system parameters and variables, respectively. The variable *il*(*t*) is a step function reflecting the given IL-1 concentration (0 or 0.000588 µM IL-1), and *uv*(*t*), *chx*(*t*) and *mg*(*t*) are step functions which are 0 if the respective stimulation is absent, and 1 if it is present.

The IKKβ phosphorylation module can be normalised without loss of generality, such that the initial conditions *ILR*(0) = 1, *ILRc*(0) = 0, *PP2A*(0) = 1 are obtained in unstimulated cells, and *IKK*(*t*) + *IKKp*(*t*) = 1 (cf. [13]). Without the weak constitutive IκBα degradation, IκBα and NF-κB would exclusively exist bound to each other in the cytosol. Also, IKK is almost completely unphosphorylated in unstimulated cells. Starting from this state, a relaxation period of 120 h before stimulation was chosen to reach steady state concentrations. Thus, *IKK*(−120 h) = 1, *IKKp*(−120 h) = 0, *IκBαNFκB*(−120 h) = *nfkb_tot_*, *NFκB_n_*(−120 h) = 0, *IκBα_t_*(−120 h) = 0, *IκBα_n_*(−120 h) = 0, *IκBα*(−120 h) = 0.

The measured IKK phosphorylation, cellular IκBα and nuclear NF-κB correspond to the observables
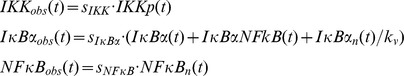



Due to the lack of an absolute reference value, the measurements of NF-κB following different stimulations were scaled with different scaling factors. A unique scaling factor was used for *IKK_obs_*, where the maximal phosphorylation is used as a reference value [13], and *IκBα_obs_*, where the initial cellular IκBα concentration was used as a reference value.

A model variant without UV-induced PP2Ac deactivation (model M_P_) was obtained setting *k_uv_ = *0. A model variant without UV-induced translational inhibition (model M_T_) was created setting *uvinh = *0.

The parameter values and boundaries were derived as follows: the IL-1 receptor turnover rate constant *k_ilr_* can be derived from [33], as shown in [34]. Depending on the number of ILR/cell (5000–15000 [33]), *k_ilr_* is between 9.3e–6 s^−1^ and 2.8e–5 s^−1^. For *k_p_*, we adopt the upper bound of 0.095 s^−1^ from [13]. The remaining parameters of the upstream module were fitted without bounds.

The association rate constant of IκBα and NF-κB, *a_1_*, was fitted within the bounds 0.3–1 µM^−1^ s^−1^ [7]. An upper bound for the parameters describing IKK-dependent degradation of free and NFκB bound IκBα, *a_2_* and *a_3_*, can also be derived: Lipniacki et al. [26] assumed an upper bound of 1 µM^−1^ s^−1^ for *a_3_*. Accounting for the IKK normalisation of the upstream module, this value has to be multiplied by the maximal IKK concentration reported in the literature, which is 0.8 µM [8], to obtain an upper bound of 0.8 s^−1^. Since *a_2_* might be equal to *a_3_* [27], we adopted the same upper bound for *a_2_*.

The degradation rate constant of IκBα mRNA, *c_3a_*, was fitted according to the parameter range allowed by different interpretations of Fig. 5 in [35]: the regression in the paper yields a half-life of about 30 min. However, half of the initial mRNA is degraded rather precisely after 45 min. On the other hand, the first 15 min may not be suitable for half-life determination since an unexpected increase of mRNA is observed. A good regression without this first data point is possible and yields a half-life of about 15 min. As a result, we choose a parameter range corresponding to IκBα mRNA half-lives from 15 to 45 min for fitting of *c_3a_*.

The upper bound of *c_4a_* was derived as shown in [26]. The parameter *c_6a_* was chosen according to [12].

For the parameter *c_5a_*, describing IKK-independent degradation of free IκBα, different values can be found in literature: O’Dea et al. [16] report a value of 0.002 s^−1^, corresponding to a half-life of about 6 min. However, experimental results may be highly variable, as apparent in measurements of Truhlar et al. (Fig. 4B and 4C in [36]): while the half-life in Fig. 4B is clearly smaller than 10 min, it is about 20 min in Fig. 4C. The same variability can be found in Mathes et al. [27]: while they report a half-life of 10 min or less, they also present pooled data from several Western blots suggesting a half-life of 15–20 min (Fig. 1B in [27]). We therefore fitted *c_5a_* within a range corresponding to half-lives between 6 and 20 min.

For *i_1a_* we adopted the fitting range as determined by Ashall et al. [12]. As in previous models (e.g. [12], [26]), *e_1a_* is assumed as 2*·i_1a_*, as can be derived from [37].

For the total cell volume, *volume*, a value of 2 pl was measured in keratinocytes [38], which corresponds to the value assumed by [26] for fibroblasts.

The ratio of cytoplasmic to nuclear volume, *k_v_*, was measured as 2.9 in keratinocytes [39].

The concentration of NF-κB if it is completely present in the cytoplasm, *nkfb_tot_*, can be calculated as
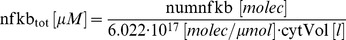
where *numnfkb*, the total number of NF-κB molecules in the cell, is 60000 molecules [40], and the volume of the cytoplasm, *cytVol*, is calculated as *volume*[l]/(1+1/*k_v_*).

Lipniacki et al. [26] derive an upper bound for *c_1a_*, c_1a,max_, as follows: based on biological considerations, they determine the maximal mRNA production rate as 0.16 molec/s and assume that this rate is reached when all NF-κB is located in the nucleus. Consequently
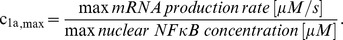



Since the mRNA production rate is counted per cytoplasmic volume and the NF-κB concentration refers to the nuclear volume, this corresponds to
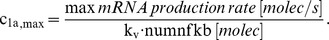



We therefore obtain a value of 9.2e–7 s^−1^ for *c_1a,max_*.

The scaling parameters are fitted without bounds. The maximal value for the UV-induced translation attenuation factor, *uvinh*, is 1 (complete inhibition).

Table S1 summarises the model parameterisation.

The MATLAB (The MathWorks) based software toolbox PottersWheel 2.0 [41] was used for the solution, optimisation and analysis of the ordinary differential equation systems. The χ^2^ value was chosen as objective function, with
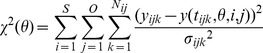
where *S* is the number of different experimental settings, *O* is the number of observables, *N_ij_* is the number of data points for observable *j* under stimulation *i*, *y_ijk_* is data point *k* of observable *j* under stimulation *i* with standard deviation *σ_ijk_*, and *y*(*t_ijk_*,*θ*,*i*,*j*) is the simulated value of observable *j* under stimulation *i* at time point *t_ijk_* for the parameter vector *θ*. The notion “χ^2^ value” is adopted from PottersWheel though a normal distribution of the errors cannot be guaranteed. However, we draw no conclusions relying on the assumption of normally distributed errors.

The parameter values of [13] and [26] were used to derive plausible initial parameter values prior to fitting. Minimisation was performed with PottersWheel’s pwF3 routine using a trust region approach. The pwF3 routine performs fits starting from the parameter values of the currently best fit randomly disturbed by a factor 10^ε^, where ε∼N(0,n). We performed 4 subsequent runs with 1000 fits each, where n = 4, 1, 0.1, 0.01, respectively, keeping the best fit of the previous run.

### II) Experimental Procedures and Data Processing

The human epithelial carcinoma cell line KB (ATCC) was cultured in RPMI 1640, 10% FCS at 37°C and 5% CO_2_. Subconfluent cells were stimulated in colourless medium with 2% FCS. Cells were stimulated with 10 ng/ml recombinant human IL-1β (R&D Systems) or co-stimulated with IL-1 and 300 J/m^2^ UVB using 6 TL12 fluorescent bulbs (290–320 nm, Philips). MG132 (25 µM, Merck) was added either 1 h prior to or 15 min after IL-1 and IL-1+UVB stimulation, depending on the specific experiment. Cycloheximide (CHX) was added at a final concentration of 5 µg/ml. At the indicated time points, the IκBα status was determined by Western blot analysis using a specific antibody (L35A5, Cell Signalling Inc). Equal loading was monitored by reprobing the membrane with an antibody directed against α-tubulin (DM1A, Neomarkers). Data from electrophoretic mobility shift analysis as well as IKKβ phosphorylation were extracted from own previously published data [25], [13].

The NF-κB time courses were scaled to a mean of 1 and subsequently averaged. IκBα Western blots were standardised to an initial value of 1. For each pooled time series, it was further assumed that the standard deviation of each value was not smaller than 10% of the maximal value of the respective pooled time series. Obvious blot artefacts were discarded. Each time series was generated from 3 independently performed experiments.

## Supporting Information

Figure S1 **Model results of model M_T_.** Experimental data and standard deviations (blue) of phosphorylated IKKβ, cellular IκBα and nuclear NF-κB are compared to the results of model M_T_ (red) fitted to these data.
(TIF)

Figure S2 **Model results of model M_P_.** Experimental data and standard deviations (blue) of phosphorylated IKKβ, cellular IκBα and nuclear NF-κB are compared to the results of model M_P_ (red) fitted to these data. The IκBα overshoot following IL-1 stimulation, the sustained low level of phosphorylated IKKβ following IL-1+UVB stimulation and the IκBα decrease following UVB stimulation are not convincingly reproduced.
(TIF)

Figure S3 **Model results of model M_T_, including IL-1+UVB+MG132 stimulation.** Experimental data and standard deviations (blue) of phosphorylated IKKβ, cellular IκBα and nuclear NF-κB are compared to the results of model M_T_ (red) fitted to these data. The IκBα overshoot following IL-1 stimulation is not reproduced.
(TIF)

Figure S4 **Model results of model M_Ref_, including IL-1+UVB+MG132 stimulation.** Experimental data and standard deviations (blue) of phosphorylated IKKβ, cellular IκBα and nuclear NF-κB are compared to the results of model M_Ref_ (red) fitted to these data.
(TIF)

Figure S5 **Model results of model M_Ref_ fitted to all experimental data simultaneously.** Experimental data and standard deviations (blue) of phosphorylated IKKβ, cellular IκBα and nuclear NF-κB are compared to the results of model M_Ref_ (red) fitted to these data.
(TIF)

Figure S6 **Relevance of altered UVB-induced translational inhibition and PP2Ac deactivation for NF-κB activity upon UVB stimulation.** This scenario considers a cell with constitutive IKKβ phosphorylation altered by a factor of 3 and PP2Ac activity altered by a factor of 0.01, compared to the reference scenario (Table S1). Knocking out the processes of UVB-induced translational inhibition or PP2Ac deactivation *in silico*, their importance for the decrease of IκBα in model M_Ref_ is determined. In contrast to the situation in the reference scenario (Figure 5B), translational inhibition alone is sufficient to induce fast IκBα degradation upon UVB stimulation.
(TIF)

Table S1 **Parameter values and descriptions.** Parameter values of model M_Ref_ fitted to all data. Note that the state variables of the IKKβ phosphorylation module are normalised and dimensionless. Parameters without fit range are fitted without bounds.
(PDF)

Table S2 **Description of the system variables and inputs.**
(PDF)

File S1 **PottersWheel model definition file for model M_Ref_**.
(M) Click here for additional data file.

## References

[1] Maeda S, Omata M (2008). Inflammation and cancer: role of nuclear factor-kappaB activation.. Cancer Sci.

[2] Naugler WE, Karin M (2008). NF-kappaB and cancer-identifying targets and mechanisms.. Curr Opin Genet Dev.

[3] Karin M, Yamamoto Y, Wang QM (2004). The IKK NF-kappa B system: a treasure trove for drug development.. Nat Rev Drug Discov.

[4] Delhase M, Hayakawa M, Chen Y, Karin M (1999). Positive and negative regulation of IkappaB kinase activity through IKKbeta subunit phosphorylation.. Science.

[5] Scott ML, Fujita T, Liou HC, Nolan GP, Baltimore D (1993). The p65 subunit of NF-kappa B regulates I kappa B by two distinct mechanisms.. Gene Dev.

[6] Cheong R, Hoffmann A, Levchenko A (2008). Understanding NF-kappaB signaling via mathematical modeling.. Mol Syst Biol 4.

[7] Hoffmann A, Levchenko A, Scott ML, Baltimore D (2002). The IkappaB-NF-kappaB signaling module: temporal control and selective gene activation.. Science.

[8] Kearns JD, Basak S, Werner SL, Huang CS, Hoffmann A (2006). IkappaBepsilon provides negative feedback to control NF-kappaB oscillations, signaling dynamics, and inflammatory gene expression.. J Cell Biol.

[9] Werner SL, Barken D, Hoffmann A (2005). Stimulus specificity of gene expression programs determined by temporal control of IKK activity.. Science.

[10] Cheong R, Bergmann A, Werner SL, Regal J, Hoffmann A (2006). Transient IkappaB kinase activity mediates temporal NF-kappaB dynamics in response to a wide range of tumor necrosis factor-alpha doses.. J Biol Chem.

[11] Cho KH, Shin SY, Lee HW, Wolkenhauer O (2003). Investigations into the analysis and modeling of the TNFalpha-mediated NF-kappaB-signaling pathway.. Genome Res.

[12] Ashall L, Horton CA, Nelson DE, Paszek P, Harper CV (2009). Pulsatile stimulation determines timing and specificity of NF-kappaB-dependent transcription.. Science.

[13] Witt J, Barisic S, Schumann E, Allgöwer F, Sawodny O (2009). Mechanism of PP2A-mediated IKK beta dephosphorylation: a systems biological approach.. BMC Syst Biol.

[14] Witt J, Barisic S, Sawodny O, Ederer M, Kulms D (2011). Modeling time delay in the NFkappaB signaling pathway following low dose IL-1 stimulation.. EURASIP J Bioinform Syst Biol.

[15] Tay S, Hughey JJ, Lee TK, Lipniacki T, Quake SR (2010). Single-cell NF-kappaB dynamics reveal digital activation and analogue information processing.. Nature.

[16] O’Dea EL, Kearns JD, Hoffmann A (2008). UV as an amplifier rather than inducer of NF-kappaB activity.. Mol Cell.

[17] Neumann L, Pforr C, Beaudouin J, Pappa A, Fricker N (2010). Dynamics within the CD95 death-inducing signaling complex decide life and death of cells.. Mol Syst Biol.

[18] Mengel B, Hunziker A, Pedersen L, Trusina A, Jensen MH (2010). Modeling oscillatory control in NF-kappaB, p53 and Wnt signaling.. Curr Opin Genet Dev.

[19] Paszek P, Ryan S, Ashall L, Sillitoe K, Harper CV (2010). Population robustness arising from cellular heterogeneity.. Proc Natl Acad Sci U S A.

[20] Pöppelmann B, Klimmek K, Strozyk E, Voss R, Schwarz T (2005). NFkappaB-dependent down-regulation of tumor necrosis factor receptor-associated proteins contributes to interleukin-1-mediated enhancement of ultraviolet B-induced apoptosis.. J Biol Chem.

[21] Kothny-Wilkes G, Kulms D, Luger TA, Kubin M, Schwarz T (1999). Interleukin-1 protects transformed keratinocytes from tumor necrosis factor-related apoptosis-inducing ligand- and CD95-induced apoptosis but not from ultraviolet radiation-induced apoptosis.. J Biol Chem.

[22] Campbell KJ, Rocha S, Perkins ND (2004). Active repression of antiapoptotic gene expression by RelA(p65) NF-kappa B. Mol Cell.

[23] Rocha S, Campbell KJ, Perkins ND (2003). p53- and Mdm2-independent repression of NF-kappa B transactivation by the ARF tumor suppressor.. Mol Cell.

[24] Strozyk E, Pöppelmann B, Schwarz T, Kulms D (2006). Differential effects of NF-kappaB on apoptosis induced by DNA-damaging agents: the type of DNA damage determines the final outcome.. Oncogene.

[25] Barisic S, Strozyk E, Peters N, Walczak H, Kulms D (2008). Identification of PP2A as a crucial regulator of the NF-kappaB feedback loop: its inhibition by UVB turns NF-kappaB into a pro-apoptotic factor.. Cell Death Differ.

[26] Lipniacki T, Paszek P, Brasier ARAR, Luxon B, Kimmel M (2004). Mathematical model of NF-kappaB regulatory module.. J Theor Biol.

[27] Mathes E, O’Dea EL, Hoffmann A, Ghosh G (2008). NF-kappaB dictates the degradation pathway of IkappaBalpha.. EMBO J.

[28] Jiang HY, Wek RC (2005). Gcn2 phosphorylation of eIF2alpha activates NF-kappaB in response to UV irradiation.. Biochem J.

[29] Wu S, Tan M, Hu Y, Wang JL, Scheuner D (2004). Ultraviolet light activates NFkappaB through translational inhibition of IkappaBalpha synthesis.. J Biol Chem.

[30] Lee EG, Boone DL, Chai S, Libby SL, Chien M (2000). Failure to regulate TNF-induced NF-kappaB and cell death responses in A20-deficient mice.. Science.

[31] Cowan JL, Morley SJ (2004). The proteasome inhibitor, MG132, promotes the reprogramming of translation in C2C12 myoblasts and facilitates the association of hsp25 with the eIF4F complex.. Eur J Biochem.

[32] Zhong JL, Yang L, Lü F, Xiao H, Xu R (2011). UVA, UVB and UVC induce differential response signaling pathways converged on the eIF2alpha phosphorylation.. Photochem Photobiol.

[33] Qwarnstrom EE, Page RC, Gillis S, Dower SK (1988). Binding, internalization, and intracellular localization of interleukin-1 beta in human diploid fibroblasts.. J Biol Chem.

[34] Witt J, Husser S, Kulms D, Barisic S, Sawodny O (2007). Modeling of IL-1 induced NF-kappaB signaling and analysis of additional UVB influence.. In: Proceedings of the SICE Annual Conference..

[35] Blattner C, Kannouche P, Litfin M, Bender K, Rahmsdorf HJ (2000). UV-induced stabilization of c-fos and other short-lived mRNAs.. Mol Cell Biol.

[36] Truhlar SME, Mathes E, Cervantes CF, Ghosh G, Komives EA (2008). Pre-folding IkappaBalpha alters control of NF-kappaB signaling.. J Mol Biol.

[37] Yang L, Chen H, Qwarnstrom E (2001). Degradation of IkappaBalpha is limited by a postphosphorylation/ubiquitination event.. Biochem Biophys Res Commun.

[38] Warskulat U, Brookmann S, Reinen A, Häussinger D (2007). Ultraviolet B radiation induces cell shrinkage and increases osmolyte transporter mRNA expression and osmolyte uptake in HaCaT keratinocytes.. Biol Chem.

[39] Schmierer B, Hill CS (2005). Kinetic analysis of Smad nucleocytoplasmic shuttling reveals a mechanism for transforming growth factor beta-dependent nuclear accumulation of Smads.. Mol Cell Biol.

[40] Carlotti F, Chapman R, Dower SK, Qwarnstrom EE (1999). Activation of nuclear factor kappaB in single living cells. Dependence of nuclear translocation and anti-apoptotic function on EGFPRELA concentration.. J Biol Chem.

[41] Maiwald T, Timmer J (2008). Dynamical modeling and multi-experiment fitting with PottersWheel.. Bioinformatics.

