# Supplementary figures and images for: Analysing the Role of UVB-Induced Translational Inhibition and PP2Ac Deactivation in NF-κB Signalling Using a Minimal Mathematical Model
Source: PLoS One. 2012 Jul 18;7(7):e40274. doi: 10.1371/journal.pone.0040274 (PMC3399864; doi:10.1371/journal.pone.0040274)

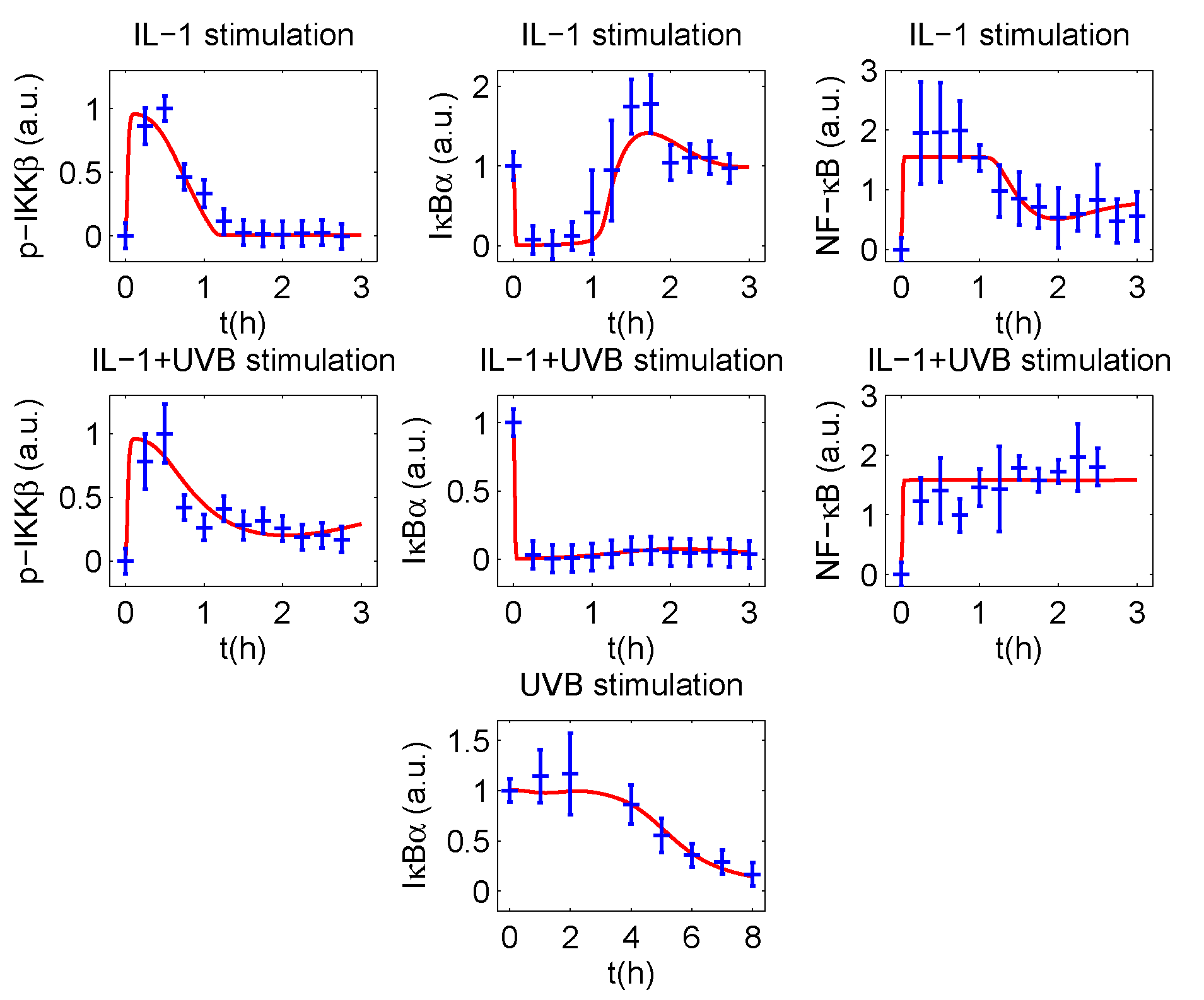

Supplement: Figure S1 — Model results of model MT. Experimental data and standard deviations (blue) of phosphorylated IKKβ, cellular IκBα and nuclear NF-κB are compared to the results of model MT (red) fitted to these data. (TIF) [file pone.0040274.s001.tif]

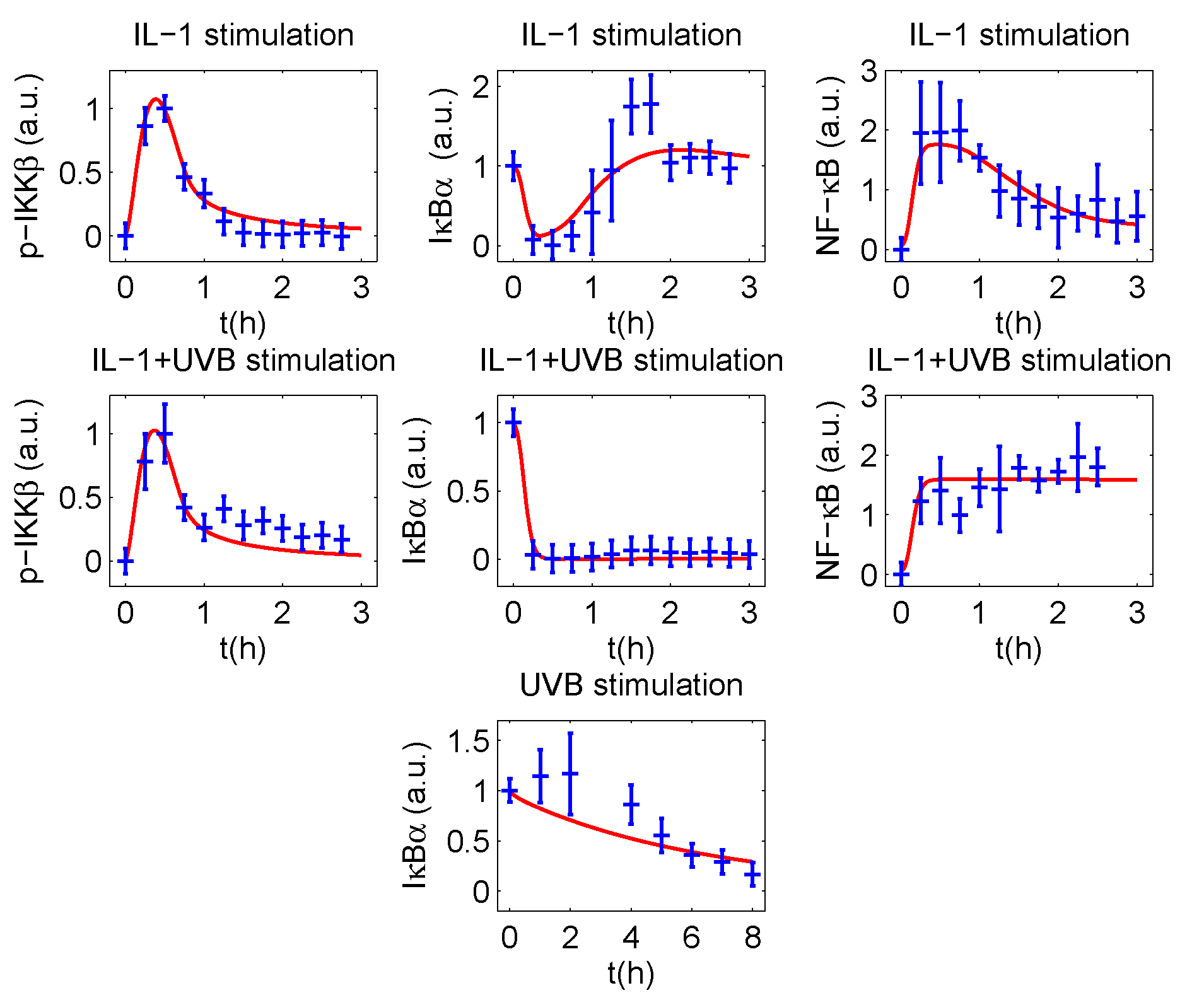

Supplement: Figure S2 — Model results of model MP. Experimental data and standard deviations (blue) of phosphorylated IKKβ, cellular IκBα and nuclear NF-κB are compared to the results of model MP (red) fitted to these data. The IκBα overshoot following IL-1 stimulation, the sustained low level of phosphorylated IKKβ following IL-1+UVB stimulation and the IκBα decrease following UVB stimulation are not convincingly reproduced. (TIF) [file pone.0040274.s002.tif]

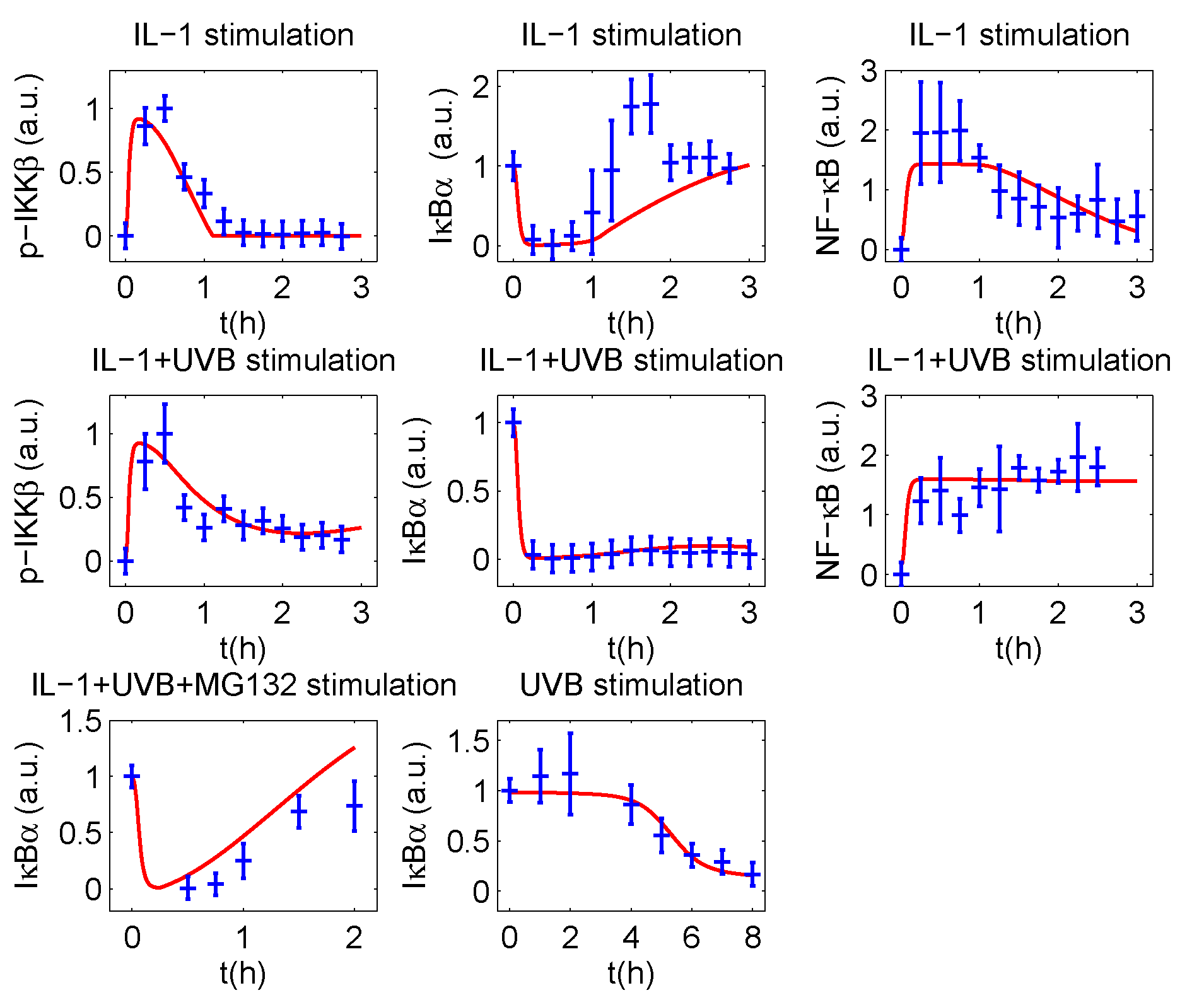

Supplement: Figure S3 — Model results of model MT, including IL-1+UVB+MG132 stimulation. Experimental data and standard deviations (blue) of phosphorylated IKKβ, cellular IκBα and nuclear NF-κB are compared to the results of model MT (red) fitted to these data. The IκBα overshoot following IL-1 stimulation is not reproduced. (TIF) [file pone.0040274.s003.tif]

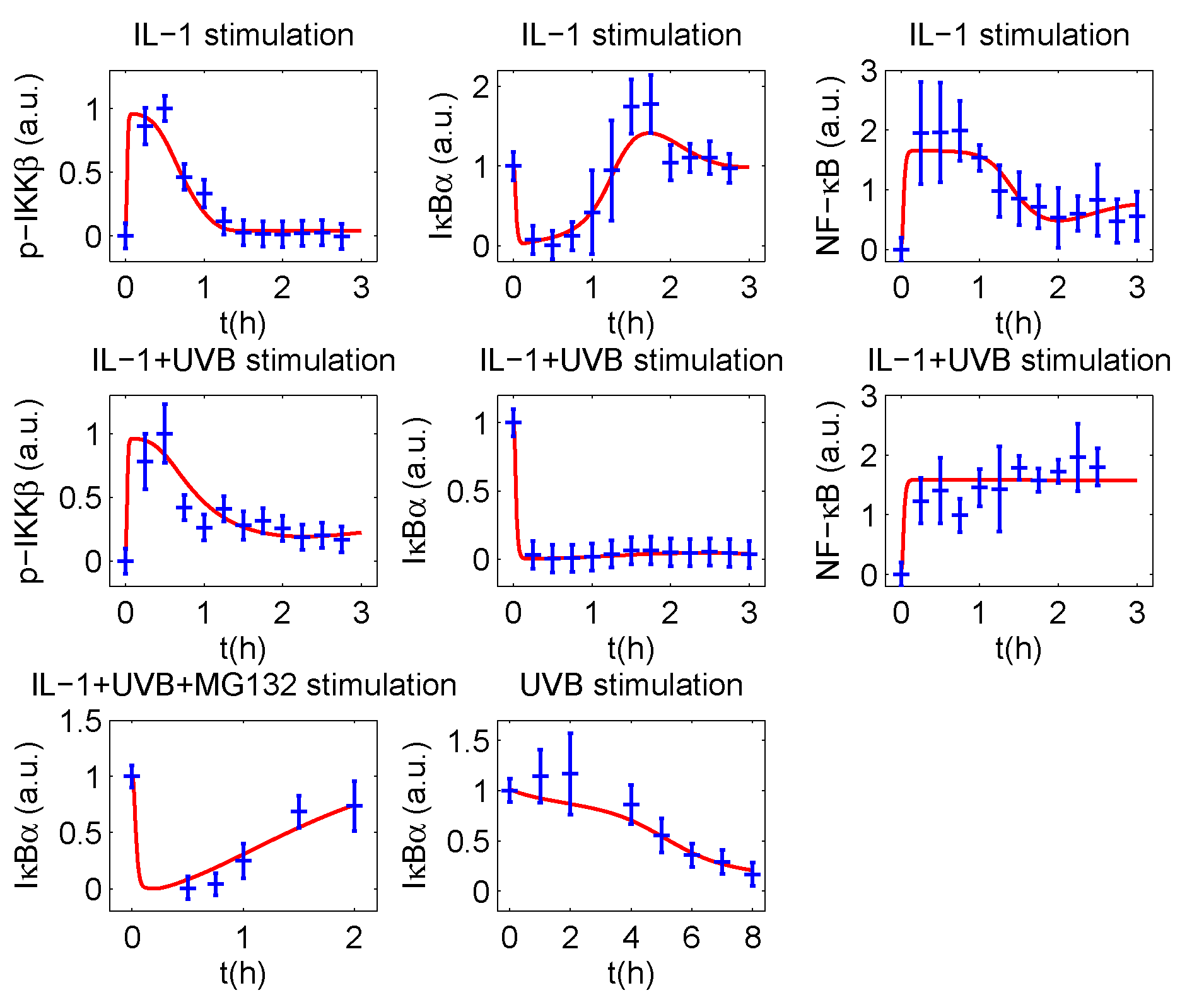

Supplement: Figure S4 — Model results of model MRef, including IL-1+UVB+MG132 stimulation. Experimental data and standard deviations (blue) of phosphorylated IKKβ, cellular IκBα and nuclear NF-κB are compared to the results of model MRef (red) fitted to these data. (TIF) [file pone.0040274.s004.tif]

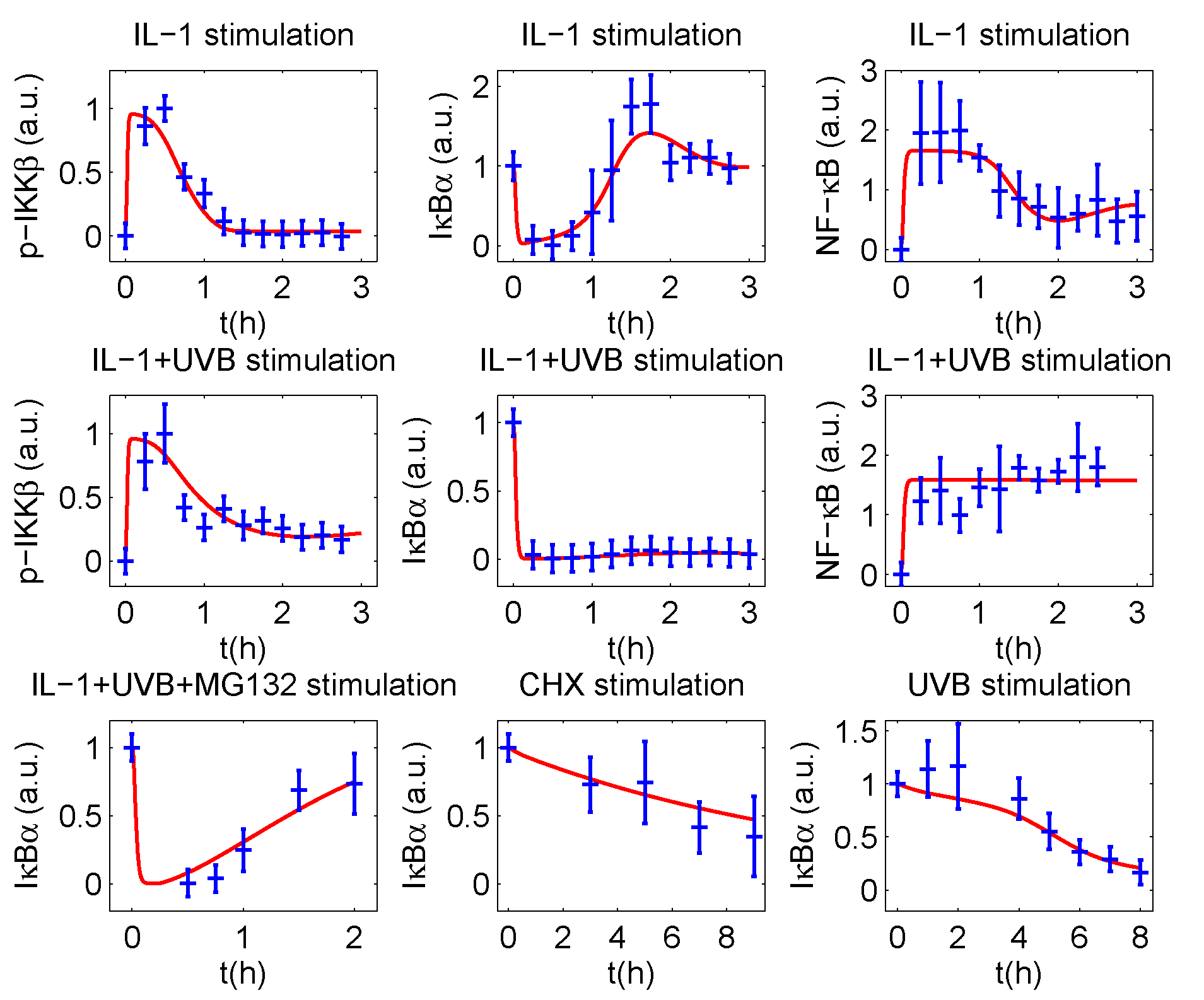

Supplement: Figure S5 — Model results of model MRef fitted to all experimental data simultaneously. Experimental data and standard deviations (blue) of phosphorylated IKKβ, cellular IκBα and nuclear NF-κB are compared to the results of model MRef (red) fitted to these data. (TIF) [file pone.0040274.s005.tif]

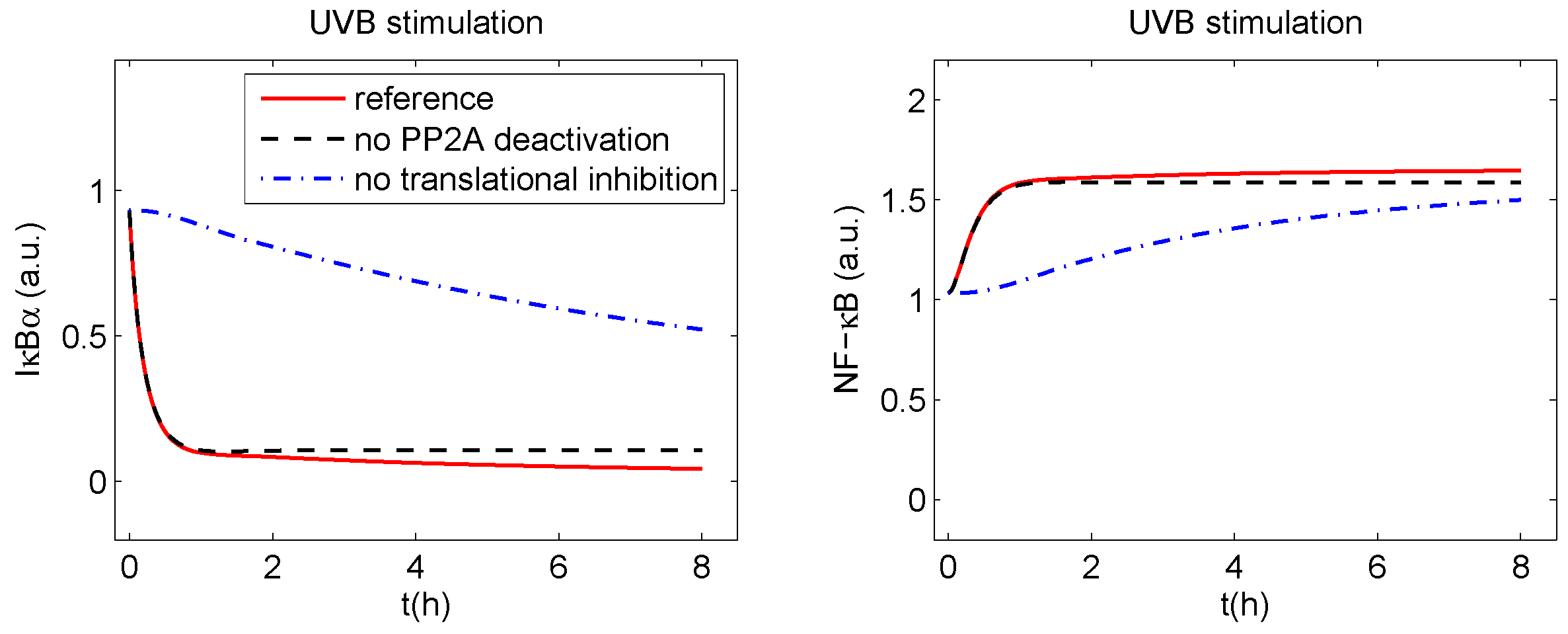

Supplement: Figure S6 — Relevance of altered UVB-induced translational inhibition and PP2Ac deactivation for NF-κB activity upon UVB stimulation. This scenario considers a cell with constitutive IKKβ phosphorylation altered by a factor of 3 and PP2Ac activity altered by a factor of 0.01, compared to the reference scenario (Table S1). Knocking out the processes of UVB-induced translational inhibition or PP2Ac deactivation in silico, their importance for the decrease of IκBα in model MRef is determined. In contrast to the situation in the reference scenario (Figure 5B), translational inhibition alone is sufficient to induce fast IκBα degradation upon UVB stimulation. (TIF) [file pone.0040274.s006.tif]
