# Supplementary material for: Analysing the Role of UVB-Induced Translational Inhibition and PP2Ac Deactivation in NF-κB Signalling Using a Minimal Mathematical Model
Source: PLoS One. 2012 Jul 18;7(7):e40274. doi: 10.1371/journal.pone.0040274 (PMC3399864; doi:10.1371/journal.pone.0040274)
Supplement: Table S1 — Parameter values and descriptions. Parameter values of model MRef fitted to all data. Note that the state variables of the IKKβ phosphorylation module are normalised and dimensionless. Parameters without fit range are fitted without bounds. (PDF) [file pone.0040274.s007.pdf]

Name	Description	Value	Unit	Fit range
k_{ilr}	ILR turnover	1.9e-5	s^{-1}	[9.3e-6, 2.8e-5]
k_a	ILR complex formation	6.5	$\mu M^{-1}s^{-1}$	-
k_i	ILR complex internalisation	0.0032	s^{-1}	-
k_p	IKK phosphorylation	0.095	s^{-1}	[0, 0.095]
k_{dp}	IKK dephosphorylation	7.1e-4	s^{-1}	-
K_m	Michaelis constant for IKK dephosphorylation	0.014	-	-
k_{uv}	UV-induced PP2A deactivation	3.6e-4	s^{-1}	-
a_1	IκBα-NFκB association	1	μM^{-1} s^{-1}	[0.3, 1]
a_2	degradation of free IκBα	0.015	s^{-1}	[0, 0.8]
a_3	degradation of NF-κB-bound IκBα	0.014	s^{-1}	[0, 0.8]
c_{1a}	IκBα mRNA transcription	9.2e-7	s^{-1}	[0, 9.2e-7]
c_{3a}	IκBα mRNA degradation	6.0e-4	s^{-1}	[2.6e-4, 7.7e-4]
c_{4a}	IκBα translation	0.5	s^{-1}	[0, 0.5]
c_{5a}	IKK-independent degradation of free IκBα	7.5e-4	s^{-1}	[5.8e-4, 2e-3]
i_{1a}	IκBα nuclear import	6.7e-4	s^{-1}	[1.9e-4, 6.7e-4]
e_{1a}	IκBα nuclear export	3.4e-4	s^{-1}	calculated as $i_{1a}/2$
$uvinh$	UVB-induced translational inhibition	0.92	-	[0, 1]
c_{6a}	constitutive IκBα degra-dation in IκBα-NF-κB complex	2.2e-5	s^{-1}	fix
k_{pconst}	constitutive IKKβ phosphorylation	2.1e-6	s^{-1}	-
k_v	volume ratio cytoplasm / nucleus	2.9	-	fix
$nfkb_{tot}$	concentration of NF-κB if completely in the cytoplasm	0.067	μM	fix
$volume$	total cell volume	2	pl	fix
s_{IKK}	IKK scaling factor	0.98	-	-
$s_{I\kappa B\alpha}$	IκBα scaling factor	14.6	-	-
$s_{NF\kappa B1}$	NF-κB scaling factor (IL-1 stimulation)	8.5	-	-
$s_{NF\kappa B2}$	NF-κB scaling factor (IL-1+UVB stimulation)	8.1	-	-
