# Supplementary material for: Analysing the Role of UVB-Induced Translational Inhibition and PP2Ac Deactivation in NF-κB Signalling Using a Minimal Mathematical Model
Source: PLoS One. 2012 Jul 18;7(7):e40274. doi: 10.1371/journal.pone.0040274 (PMC3399864; doi:10.1371/journal.pone.0040274)
Supplement: Table S2 — Description of the system variables and inputs. (PDF) [file pone.0040274.s008.pdf]

**variable**	**description**
$ILR(t)$	IL-1 receptor (normed)
$ILRc(t)$	IL-1 receptor complex (normed)
$IKK(t)$	unphosphorylated IKK (normed)
$IKKp(t)$	phosphorylated IKK (normed)
$PP2A(t)$	active PP2A (normed)
$I\kappa B\alpha NF\kappa B(t)$	cytosolic IκBα-NF-κB complex
$I\kappa B\alpha(t)$	free cytosolic IκBα
$I\kappa B\alpha_n(t)$	free nuclear IκBα
$NF\kappa B_n(t)$	free nuclear NF-κB
$I\kappa B\alpha_t(t)$	IκBα mRNA
**input**	
$il(t)$	interleukin-1β
$uv(t)$	UVB irradiation
$mg(t)$	MG132
$chx(t)$	cycloheximide
